# Cluster-based exposure variation analysis

**DOI:** 10.1186/1471-2288-13-54

**Published:** 2013-04-04

**Authors:** Afshin Samani, Svend Erik Mathiassen, Pascal Madeleine

**Affiliations:** 1 Laboratory for Ergonomics and Work-related Disorders, Center for Sensory-Motor Interaction (SMI), Department of Health Science and Technology, Aalborg University, Fredrik Bajers Vej 7 D-3, Aalborg East 9220, Denmark; 2 Centre for Musculoskeletal Research, Department of Occupational and Public Health Sciences, Faculty of Health and Occupational Studies, University of Gävle, SE 801 76, Gävle, Sweden

**Keywords:** Ergonomics, Physical work load, Linear discriminant analysis, Work-related musculoskeletal disorders, Principle component analysis

## Abstract

**Background:**

Static posture, repetitive movements and lack of physical variation are known risk factors for work-related musculoskeletal disorders, and thus needs to be properly assessed in occupational studies. The aims of this study were (i) to investigate the effectiveness of a conventional exposure variation analysis (EVA) in discriminating exposure time lines and (ii) to compare it with a new cluster-based method for analysis of exposure variation.

**Methods:**

For this purpose, we simulated a repeated cyclic exposure varying within each cycle between “*low*” and “*high*” exposure levels in a “*near*” or “*far*” range, and with “*low*” or “*high*” velocities (exposure change rates). The duration of each cycle was also manipulated by selecting a “*small*” or “*large*” standard deviation of the cycle time. Theses parameters reflected three dimensions of exposure variation, i.e. range, frequency and temporal similarity.

Each simulation trace included two realizations of 100 concatenated cycles with either low (ρ = 0.1), medium (ρ = 0.5) or high (ρ = 0.9) correlation between the realizations. These traces were analyzed by conventional EVA, and a novel cluster-based EVA (C-EVA). Principal component analysis (PCA) was applied on the marginal distributions of 1) the EVA of each of the realizations (univariate approach), 2) a combination of the EVA of both realizations (multivariate approach) and 3) C-EVA. The least number of principal components describing more than 90% of variability in each case was selected and the projection of marginal distributions along the selected principal component was calculated. A linear classifier was then applied to these projections to discriminate between the simulated exposure patterns, and the accuracy of classified realizations was determined.

**Results:**

C-EVA classified exposures more correctly than univariate and multivariate EVA approaches; classification accuracy was 49%, 47% and 52% for EVA (univariate and multivariate), and C-EVA, respectively (p < 0.001). All three methods performed poorly in discriminating exposure patterns differing with respect to the variability in cycle time duration.

**Conclusion:**

While C-EVA had a higher accuracy than conventional EVA, both failed to detect differences in temporal similarity. The data-driven optimality of data reduction and the capability of handling multiple exposure time lines in a single analysis are the advantages of the C-EVA.

## Background

Work-related musculoskeletal disorders (WMSD) are major problems in the industrialized world, at the individual, company and societal levels [1]. Risk factors of WMSD are multidimensional in the sense that individual, physical as well as psychosocial factors play a role in the development of these disorders [2]. Constrained postures, repetitive movements, heavy manual handling, lack of variation and insufficient recovery, are often cited as physical risk factors [3]. The extent and structure of variation of biomechanical exposure across time is generally accepted to be an important determinant of the risk of contracting WMSD [4]. Thus, it has been hypothesized that increased exposure variation may prevent WMSD development in jobs characterized by long-term exposure to constrained postures and/or repetitive movements [5-7]. This calls for the development of methods that can quantify biomechanical exposure variation within and between subjects at work [7,8].

Biomechanical exposure in occupational settings has been described to comprise three basic, conceptual dimensions, i.e., level (amplitude), duration and repetitiveness (frequency), the latter being closely associated with velocity and acceleration when postural exposure is of interest [9,10]. Exposure “variation”, in turn, has been defined as the change in the exposure over time [7], including three basic aspects: the range of exposure changes, the repetitiveness (frequency) of those changes, and the extent of temporal similarities, or recurring patterns, in the exposure time line.

A computational framework, the exposure variation analysis (EVA), has been suggested to quantify variation [11]. EVA quantifies the accumulated proportion of recorded time that the exposure level remains uninterruptedly within pre-determined limits (“exposure level” categories) for pre-determined periods of time (“sequence duration” categories). In ergonomics studies, EVA has mainly been applied with recordings of postures (e.g. [6,12,13]) and electromyography (e.g. [14-16]). A variety of metrics have been suggested to assess differences in EVA results between groups or conditions and to extract what is believed to be important properties of exposure. Some approaches are based on an aggregation of EVA cells below or above a certain threshold in exposure level and/or sequence duration [12,14,17], while others derive variables describing the centroid or standard deviation of the EVA cells [13,16,18,19], or suggest statistical analysis procedures using principal component analysis of the EVA marginal distribution [15] and hierarchical regression of exposure level, frequency and duration simultaneously [20].

In most studies using EVA, the boundaries of the exposure level categories are based on a logarithmic lay-out [11], but equidistant [20] and non-uniform layouts [12] have also been suggested. A non-equidistant lay-out of EVA may cause a correlation between exposure level and “sequence duration” categories in some cases [21], which may violate the intention of EVA to state the “true” interaction between the independent aspects of level and frequency in an exposure time line. A data-driven approach for classifying exposures may organize the data in the EVA array preserving the aforementioned independence optimally. This may, in turn, increase the ability of the approach to discriminate different time lines of exposure compared to a conventional EVA, and thus, eventually, classify exposure patterns according to variation and, possibly, risk.

The conventional EVA is univariate, in the sense that it addresses one exposure time line at a time. If a set of outcomes is correlated, for instance a number of EVA analyses of different exposure variables during the same work task, repeated univariate analyses may increase the risk of type I error in a statistical inference [22].

Using statistical simulations, it is possible to generate synthetic exposure time lines mimicking lack or excess of exposure variation. This allows for a comparison of different methods for exposure variation quantification in terms of their ability to pick up aspects of the variation. This approach was applied in the present study, in which we develop a data driven analysis approach based on conventional EVA to quantify variation in exposure and investigate whether this new approach can lead to a better performance in discriminating between different time lines of exposure compared with a univariate and multivariate conventional EVA.

## Methods

We developed a novel data-driven exposure analysis approach based on data clustering techniques (C-EVA), and investigated its ability to discriminate between different simulated time lines of exposure compared with a conventional EVA using both univariate and multivariate approaches. Particularly, the effectiveness of the different approaches was compared in terms of their capability to handle correlated exposure realizations.

### Exposure simulation

An effective method for quantifying exposure variation should be able to discriminate exposure time lines with different properties along all three fundamental aspects (dimensions) of variation: (i) range, (ii) frequency and (iii) temporal similarity [7]. Thus we simulated the extent of exposure variation in a cyclic movement by two sets of parameters representing “small” and “large” exposure variation along each of these dimensions to investigate the discriminative ability.

**Figure 1:**
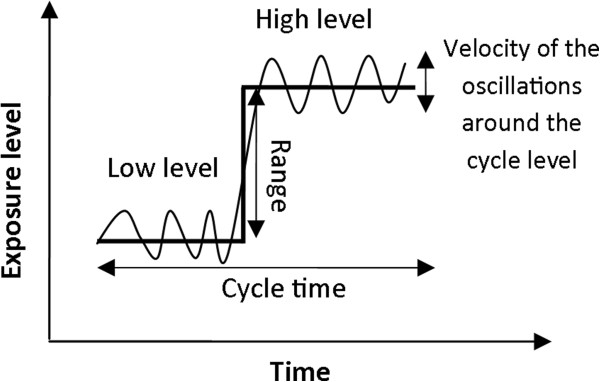
**Illustration of one template cycle and the simulated variables.** Two levels of input parameters were applied for each of the three exposure dimensions, i.e., range, velocity and cycle time standard deviation. The thick line represents the exposure average at the low and high exposure level; in the simulated cycles exposure varied around this level as indicated by the waveforms.

Cyclic movements represent a repetitive exposure pattern which offers a conceptual base for investigating metrics for exposure variation. In our simulation, a cycle was composed of two successive levels of exposure, one “high” and one “low” (see Figure 1). The high exposure level was obtained by adding a simulated “range” to the low exposure level. The simulation design rendered 2x2x2 exposure groups representing different sizes of range (“far”, “near”), velocity (“high”, “low”) of exposure shifts around the average level, and temporal similarity (“large”, “small”) of exposure sequences between cycles.

Parameter settings were retrieved from literature on right upper arm postural exposure in cyclic occupational work due to its relevance to WMSD in the neck and shoulder region [23]. For all exposure groups, the ratio of low to high level duration (duty cycle) was randomly selected within a narrow range (0.6-0.7). The mean cycle time was set to be equal in all groups but its standard deviation was allowed to change, with a large and a small variability representing different extents of “temporal similarity” between the cycles. Table 1 summarizes the chosen parameter settings in the simulation.

**Table 1:** Statistical descriptors of the exposure variation dimensions (level, repetitiveness/velocity and similarity), with parameter values used in the simulation

**Cycle parameters**	**Size of variation**	**Statistical descriptors**	**Values in the literature**	**Applied distribution**
Level	low	mean	25° ^*^	N(25,4.5^2^)
		SD	4.5° ^*^	
	high	10^th^	33° ^#^	N(45,7.5^2^)
		90^th^	53°^#^	
Velocity	low	50^th^	3°/s ^$^	log-N(1.1,1.3^2^)
		90^th^	16°/s ^$^	
	high	50^th^	38°/s ^#^	log-N(3.6,0.9^2^)
		90^th^	122°/s ^#^	
Duration	small	mean	216 s ^£^	N(216, 36.3^2^)
		SD	36.3 s ^£^	
	large	mean	216 s ^£^	N(216, 51.8^2^)
		SD	51.8 s ^£^	

The parameters of applied distributions used in the simulation of exposure variation dimensions (level, repetitiveness/velocity and similarity).

Exposure groups were simulated based on distributions parameters as shown by N(μ,σ^2^) representing a normal and log-N(μ,σ^2^) a log-normal distribution. SD stands for standard deviation and 10th, 50th and 90th indicate percentiles of the exposure distributions. The parameters of the distribution are extracted from relevant literature; ^*^, ^#^, ^$^ and ^£^ represent the studies in the literature upon which the parameters of the distributions are based. ^*^: Nordander et al (2008), ^#^: Hansson et al (2010), ^$^: Arvidsson et al (2006) and ^£^: Möller et al (2004). We refer to the reference list for detailed information.

Exposure level and cycle time duration were assumed to be normally distributed whereas a log-normal distribution was assumed for the velocity (see below). Both normal and log-normal distributions can be fully described by two parameters, the mean and the standard deviation. Thus, if statistical descriptors such as the median and the 90^th^ percentile are available, the parameters of the distribution can be derived numerically.

We simulated 100 sequential cycles and assumed that exposure was recorded at a virtual sampling frequency of 20 Hz [24]. The whole sequence composed of concatenation of100 simulated cycles was termed an “*exposure realization*”. An *“exposure trace”* was defined as an entity consisting of two *exposure realizations*. For each exposure group, 30 simulated traces were obtained at each of three levels of cross-correlation coefficient between the two *exposure realizations* in an *exposure trace*: low (ρ = 0.1), medium (ρ = 0.5) and high (ρ = 0.9) [25].

### Range

As illustrated in Figure 1, the range corresponded to the difference between the “low” and “high” exposure levels within a cycle. Thus, we estimated the “high” level of each simulated cycle as the sum of the “low” level and the exposure range corresponding to the simulated scenario: “far” or “near”. We also assumed the exposure range and the low level of the cycle to be independent, so that $\sigma_{\mathrm{High}}^{2}=\sigma_{\mathrm{Low}}^{2}+\sigma_{\mathrm{Range}}^{2}$. The “near” range was assumed to have a mean and standard deviation roughly one third of that of the “far” range. Thus, the distribution of “near” and “far” range were obtained normally distributed as N(7,2^2^) and N(20,6^2^) respectively.

### Velocity/frequency

Since the reported descriptors in the literature indicate a skewed velocity distribution, we assumed that the velocity distribution can be approximated by a log-normal distribution. In general, the literature reports absolute values of angular velocity [24,26]. Thus, the extracted parameters correspond to the distribution of absolute value of angular velocity.

To control the velocity direction (up/down), half of the randomly generated samples were randomly selected and their signs were toggled (if *y* = |*x*| then *f*_*x*_(*x*) = 0.5(*f*_*y*_(*x*) + *f*_*y*_(−*x*)) where *f*_*x*_*(x)* represents the probability density function of x.). Only half of the samples were toggled to generate a sequence close to a zero-mean process and not to introduce any net displacement after the numerical integration. The velocity traces were filtered, i.e. F_cut-off_ = 5 Hz, Butterworth second order in order to keep a sensible spectrum content for simulated realizations [27]. The maximum allowed velocity was set to 300°/s [28]. The velocity traces were numerically integrated and hypothetical limits (−35° to180° mimicking anatomical constraints) were imposed to the output of integration in order to preserve the realism of resulting postures. The mean of the integration output was subtracted from the output to assure that the integration of the velocity traces would result in a zero-mean process.

### Temporal similarity

A study by Möller et al. [6] reported the mean cycle time in an assembly task to be 216 s. While both higher and lower values for cycle durations have been reported in the literature, we chose the values from this paper [6] as a realistic estimation of cycle time. The standard deviation of the cycle time varied from “small” to “large” values according to Table 1. “Small” standard deviation of cycle time reflects a cyclic task in which the cycles temporal similarity is high whereas “large” values reflect a cyclic task with low temporal similarity. Within an *exposure trace*, temporal similarity was kept identical between the two *exposure realizations*.

### Exposure variation analysis

*Exposure traces* were analyzed using the new C-EVA (see below), and the results were compared to those from the conventional EVA with predetermined exposure categories using both univariate and multivariate approaches.

### Conventional EVA with predetermined exposure categories

EVA categories were constructed using intervals of {<−20 -10 10 20 45 75 105 135°<} and {<0.7 1.5 3.1 6.3 s<} along the exposure axis (exposure level categories) and frequency axis (sequence duration categories), respectively. The exposure level categories in the EVA were set arbitrarily to avoid a too coarse or a too fine EVA array, and are similar to the set-up in several studies of working postures [12,20,29]. The “sequence duration” categories were set using an increasing width of the sequence with increasing average duration as also used in several posture studies [29]. The EVA array of each *exposure realization* was denoted as {*E*_*i*_ : *n* × *m*},  *i* = 1,  2, with *n* and *m* set to 9 and 5, respectively. The marginal distributions of EVA with respect to level and frequency were computed by adding up cell values along both dimensions [14,15]. Thus, the marginal distribution of EVA had 9 and 5 entries, corresponding to the number of categories. In total, this resulted in a 14-dimensional space as the basis for a PCA analysis. Thus, the marginal distribution was denoted as {*M*_*i*_ : 1 × (*n* + *m*)},  *i* = 1,  2.

### Cluster-based EVA

A number of methods (e.g. Hartigan and Silhouette statistics) are available for identifying the optimal number of clusters in a general multi-dimensional space [30]. Conventionally, the optimal number of clusters is defined on the basis of the within-cluster dispersion index (sum of squared deviations within clusters) versus the number of clusters [31,32]. When the number of clusters is increased, the dispersion index first decreases and then flattens markedly. The least number of clusters which leads to a flattened dispersion index defines the optimal number of clusters. *“Gap analysis”* is a formalized way to implement this procedure. “*Gap analysis*” can be applied to any clustering method such as K-means [33] to obtain the number and centers of optimal clusters [30].

A “*Gap analysis*” was performed on the exposure level of an arbitrarily selected *exposure trace* (representative *exposure trace*) from each of the exposure groups (n = 8) combined with each of the cross-correlation levels (n = 3), i.e., for 24 representative traces. Subsequently, the following procedure was carried out only once to obtain the optimal cluster centers. Thus, in terms of computational complexity, the following procedure adds just a one-time overhead to the conventional EVA.

i) Using the gap analysis, the optimal number and centers of clusters was obtained for each of the 24 representative traces.

ii) Once the centers of clusters were defined for a trace, the samples of the representative *exposure traces* were appointed to their closest cluster. Each sample (a 2 x1 vector consisting to two individual points across the time axis from the two *exposure realizations*) of an *exposure trace* would be appointed to the cluster whose center had the minimum Euclidean distance to that sample.

iii) Once the steps (i and ii) were done for all eight exposure groups at three cross-correlation level, the obtained centers of the clusters were compared and nearby clusters were merged into one cluster. Nearby clusters were defined as those having a center-to-center distance less than the 10th percentile of the center-to-center distances of all possible cluster pairs.

iv) The center of a merged cluster was defined by taking a weighted average of the centers of the constituent clusters. The weighting factor for each cluster was defined by the number of samples appointed to that cluster divided by the total number of samples appointed to the merged cluster. This procedure was repeated for all merged clusters.

v) The centers of optimal clusters for exposure level were obtained after finishing step (iv). The number of optimal clusters was denoted as *n*_*c*_. The Euclidean distance of all samples of a representative *exposure trace* to the optimal cluster centers was computed. The samples of a representative *exposure trace* were appointed to the cluster with the closest center. Thus, each sample of a representative *exposure trace* was tagged with its closest cluster. This step was repeated for all representative *exposure traces.*

vi) For each of the representative exposure traces in step (v), a sequence of cluster tags was obtained. We registered the duration of uninterrupted period in which the samples of a representative *exposure trace* tagged with one cluster. This made a sequence including durations of such periods. This was repeated for all representative exposure traces.

vii) As for exposure level, the *gap analysis* algorithm was applied to the duration of uninterrupted periods registered in the previous step. The optimal cluster centers along the sequence duration were found (in this case, the clusters are defined in 1D space - centers of an interval). The steps (ii-iv) were repeated for sequence duration to find the optimal set-up of categories. The number of optimal categories was denoted by *m*_*c*_.

Thus, the output of C-EVA quantifies the proportion of total recording time that an *exposure trace* remains uninterruptedly close to one of the optimal clusters for a certain period of time. Similar to conventional EVA, this period was partitioned into sequence duration categories in step (vii). The marginal distributions of C-EVA were also computed similar to those of conventional EVA and denoted by Mc:1×nc+mc.

### Statistics

The performances of conventional EVA and C-EVA to discriminate between the exposure groups were assessed. The performance of conventional EVA was tested using multivariate and univariate approaches as described below.

Each *exposure trace* was represented by two sets of 14 variable vectors {*M*_*i*_} *i* = 1,  2 expressing the marginal distributions of EVA. In the multivariate approach, these two vectors of the parallel traces were represented by a 28 dimensional vector {*M* = [*M*_1_ *M*_2_]}. From each exposure group and each level of cross-correlation, 20 *exposure traces* were randomly selected and used as a training set. The remaining 10 *exposure traces* constituted the test set (see below). Thus, the training set consisted of 8 × 3 × 20 *exposure traces* and the test set consisted of 8 × 3 × 10 *exposure traces*. The training set mean along each of the dimensions of {*M*_*i*_} was subtracted from both the training and test sets and the result was normalized to the standard deviation of the training set along the same dimension (taking the z-score without using the information from the test set). A principal component analysis (PCA) was performed on the training set. Each principal component describes a percentage of the total variance in the data set. The least number of principal components leading to a total sum of explained variance above 90% were kept as significant components (denoted as {*PC*_*M*_}).

In the univariate approach, a similar procedure was performed for the marginal distribution of EVA from each of the *exposure realization*. Finally, each *exposure trace* was represented by using the significant components taken from each of the *exposure traces* {[PC_M1_ PC_M2_]}. For the C-EVA, each *exposure trace* was represented by a *n*_*c*_ *+ m*_*c*_ dimensional vector {*Mc*}. Thus, PCA was applied on the training set with *n*_*c*_ *+ m*_*c*_ dimension.

For all approaches, PCA only applied on the training set and the test set was projected along significant components. This was to avoid the test set from affecting the significant components.

A linear classifier was trained using the significant components from the training set and the projected test set was utilized to validate the performance of the classification. The accuracy of the classification was averaged across 30 repetitions of randomly chosen training and test sets. The accuracy was defined as the percentage of exposure traces in the test data set which were classified to their correct exposure groups. A one-way analysis of variance (ANOVA) was used to assess the accuracy of the classification using the three approaches, i.e., EVA (univariate and multivariate) and C-EVA. Tukey's honestly significant difference criterion was applied as a post hoc test in case of the one-way ANOVA showing a significant main effect. The classification accuracy is reported with the mean (standard deviation) of accuracy in percentage. The level of significance was set to P < 0.05.

## Results

**Figure 2:**
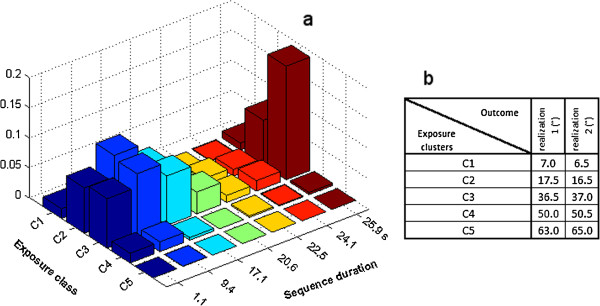
**a) The layout of the performed cluster-based exposure variation analysis.** Each bar indicates the proportion of recording time spent uninterruptedly at the indicated optimal exposure level clusters (extracted from the gap analysis procedure explained in the text) for the duration indicated by the sequence duration category. The higher the bar the longer the proportional time the samples of an exposure trace will stay close to the corresponding cluster centers. **b**) The location of the exposure level cluster centers for each of two exposure realizations.

The accuracy of classifications was 47% (SD = 4.4%), 49% (4.3%) and 52% (4.9%) for the marginal distributions of EVA (multivariate and univariate) and C-EVA, respectively. Thus, the C-EVA improved the classification accuracy slightly, but significantly (P < 0.001). Figure 2a shows an example of C-EVA performed on an *exposure trace* from an exposure group with high temporal similarity, near range and low velocity. Figure 2b illustrates the location of optimal cluster center in terms of exposure level of realization one and two. Table 2 reports in details the misclassification rates for each exposure group when using the marginal distributions of the three analysis approaches. Note that the gross misclassification rate is equal to the summation of the reported rates in Table 2 divided by number of groups (in this case 8). The most pronounced misclassification for all the three approaches occurred along the aspect of temporal similarity, i.e., the cycle time standard deviation.

**Table 2:** **Misclassification rates (Mean (SD) %) of cluster based exposure variation analysis (C-EVA) and univariate and multivariate exposure variation analysis (EVA**_**U**_**, EVA**_**M **_**respectively)**

				**Assigned exposure group**							
			**Temporal similarity:**	** Small**				** Large**			
			**Range:**	** Near**		** Far**		** Near**		** Far**	
			**velocity:**	** Low**	** High**	** Low**	** High**	** Low**	** High**	** Low**	** High**
**True exposure group**			**Approach**								
small	near	low	C-EVA	NA	0 (0)	0 (0)	0 (0)	52 (7)	0 (0)	0 (0)	0 (0)
			EVA_U_	NA	0 (0)	0 (0)	0 (0)	50 (8)	0 (0)	0 (0)	0 (0)
			EVA_M_	NA	0 (0)	0 (0)	0 (0)	48 (9)	0 (0)	0 (0)	0 (0)
small	near	high	C-EVA	0 (0)	NA	0 (0)	0 (0)	0 (0)	51 (9)	0 (0)	0 (0)
			EVA_U_	0 (0)	NA	0 (0)	0 (0)	0 (0)	47 (9)	0 (0)	0 (0)
			EVA_M_	0 (0)	NA	0 (0)	2 (2)	0 (0)	44 (10)	0 (0)	2 (3)
small	far	low	C-EVA	0 (0)	0 (0)	NA	0 (0)	0 (0)	0 (0)	45 (9)	0 (0)
			EVA_U_	0 (0)	0 (0)	NA	0 (0)	3 (1)	0 (0)	48 (9)	0 (0)
			EVA_M_	0 (0)	0 (0)	NA	0 (0)	3 (1)	0 (0)	50 (9)	0 (0)
small	far	high	C-EVA	0 (0)	0 (0)	0 (0)	NA	0 (0)	0 (0)	0 (0)	53 (9)
			EVA_U_	0 (0)	0 (0)	0 (0)	NA	0 (0)	0 (0)	0 (0)	56 (8)
			EVA_M_	0 (0)	3 (3)	0 (0)	NA	0 (0)	3 (3)	0 (0)	44 (9)
large	near	low	C-EVA	48 (9)	0 (0)	0 (0)	0 (0)	NA	0 (0)	0 (0)	0 (0)
			EVA_U_	49 (9)	0 (0)	0 (0)	0 (0)	NA	0 (0)	0 (0)	0 (0)
			EVA_M_	48 (8)	0 (0)	0 (0)	0 (0)	NA	0 (0)	0 (0)	0 (0)
large	near	high	C-EVA	0 (0)	43 (7)	0 (0)	0 (1)	0 (0)	NA	0 (0)	0 (1)
			EVA_U_	0 (0)	52 (8)	0 (0)	0 (0)	0 (0)	NA	0 (0)	4 (1)
			EVA_M_	0 (0)	50 (10)	0 (0)	5 (3)	0 (0)	NA	0 (0)	4 (3)
large	far	low	C-EVA	0 (0)	0 (0)	47 (8)	0 (0)	0 (0)	0 (0)	NA	0 (0)
			EVA_U_	0 (0)	0 (0)	51 (8)	0 (0)	0 (0)	0 (0)	NA	0 (0)
			EVA_M_	0 (0)	0 (0)	49 (9)	0 (0)	0 (0)	0 (0)	NA	0 (0)
large	far	high	C-EVA	0 (0)	0 (0)	0 (0)	44 (8)	0 (0)	0 (0)	0 (0)	NA
			EVA_U_	0 (0)	0 (0)	0 (0)	56 (9)	0 (0)	0 (0)	0 (0)	NA
			EVA_M_	0 (0)	4 (3)	0 (0)	52 (10)	0 (0)	5 (3)	0 (0)	NA

Table rows represent the true groups of the test data and columns indicate the assigned group label according to adopted approach. Percentage of misclassified samples refers to the true number of samples within each group. “small” and “large” refer to the cycle time standard deviation, “near” and “far” to the exposure range, and “low” and “high” to the movement velocity (cf. Figure 1, Table 1). NA: not applicable.

**Figure 3:**
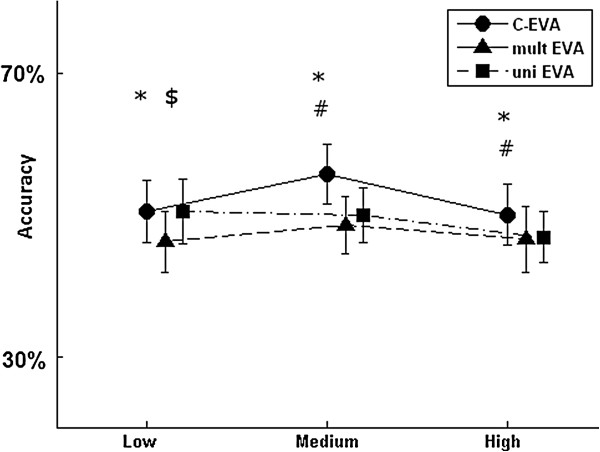
**The mean and standard deviation of classification accuracy of the cluster based exposure variation analysis (C-EVA), the multivariate exposure variation analysis approach (EVA**_**M**_**) and the univariate EVA approach (EVA**_**U**_**) at different levels of cross-correlation (Low (ρ = 0.1), Medium (ρ = 0.5) and High (ρ = 0.9)) between parallel exposure realizations in a cycle.** *, #, $; significant difference (pairwise comparison; p < 0.05) between C-EVA and EVA_M_, C-EVA and EVA_U_, and EVA_M_ and EVA_u_ respectively.

The accuracy of the classification was also checked at different levels of cross-correlation (e.g. low, medium and high) between the *exposure realizations*. Figure 3 depicts the misclassification rate at different cross-correlation level. C-EVA slightly outperformed both approaches (p < 0.001 where significant). However at low cross-correlation level, C-EVA only outperformed the multivariate approach (49.2 (3.7) % versus 46.4 (3.9) %). The univariate approach outperformed the multivariate approach at the low cross-correlation level (49.3 (3.8) % versus 46.4 (3.9) %).

## Discussion

In this study, we developed a new method, cluster-based exposure variation analysis (C-EVA) to analyze exposure variation during repeated cyclic movements. The method is based on the conventional EVA but improved its performance in identifying exposure groups that were known to differ with respect to exposure variation. We found that discriminating between exposures differing only in temporal similarity was the main challenge of all the applied methods.

### Simulation

The current simulations imitate the distributional properties of reported *exposure traces* from different repetitive occupational tasks. Since the simulations are, by necessity, simplified representations of the exposure structure of real work, they will not fully reflect all aspects of human movements during repetitive tasks. For simulating exposure velocities (change rates), log-normal distributions were selected, inspired by kinematic theory of rapid human movement [34,35], which suggest a logarithmic mathematical model for describing the velocity profile of human ballistic movements. Moreover, the log-normal distribution also matches with a positive skewness (“long tails”) of velocity profiles (10^th^, 50^th^ and 90^th^ percentiles) often reported in the literature on movements in occupational tasks, see e.g., [36,37].

Applying Bayesian networks seems, at a first glance, to be a viable approach for estimating the parameter of simulation, but it requires that the observations are delineated by a directed acyclic model [38]. Even with a successful formulating of such a model, this approach will only be applicable to model specific problems and does not provide a general computational framework.

For the ease of simulation, a few assumptions were made that may have introduced some bias. For example, the “*low*” and “*high*” exposure levels of each simulated cycle were considered to be independent, and the velocity distributions were assumed to be identical for these two levels. Additionally, the extent of exposure level were in effect smaller in “*low*” than in “*high*” velocity groups due to the combined effect of a slower changing exposure level and a similar cycle time duration. As a remedy, a correlation coefficient could be assumed between the “*low*” level of the cycles and their ranges, and the sensitivity of the performance of the approaches could have been assessed with respect to this parameter as well. Generally, since C-EVA is a data driven approach, assessment of its sensitivity to the simulation parameters settings and/or the basic simulation models is of utmost importance. Addressing these issues is an important challenge in future studies of C-EVA.

The simulation consisted of two correlated realizations of an *exposure trace*. This was a measure to decrease the risk of type I error in a statistical inference as a result of multiple univariate analyses [22]. Nevertheless, all three applied methods for analyzing variation were handling the realizations using a multivariate framework (PCA and the linear classifier). Otherwise, a decline in the rate of classification accuracy would have been trivial considering that the classification had been applied for *exposure realizations* separately (an accurate classification in this case requires two separate classification procedure for each of the exposure realizations and both procedures must identify each of the realizations in the true exposure group).

Our results showed a better performance for the univariate EVA compared with the multivariate EVA at low cross-correlation between the *exposure realizations*. This can be explained by the fact that $PC_{M}{}_{{}_{1}}$ and $PC_{M_{2}}$ are not necessarily orthogonal, so that their combination may have described more than 90% of total variance in the data set.

Applying classification analysis on EVA and C-EVA outcomes failed to discriminate simulated exposure groups differing in the temporal similarity dimension of exposure variation. Probably, more advanced methods are needed to handle this aspect of exposure variation; for example, recurrent map analysis [39] may provide a basis for performing additional analysis. Future studies are needed, devoted to effective procedures for discrimination of temporal similarity, including validating these procedures on experimental data sets.

### C-EVA advantages and drawbacks

A general issue in density estimation using constant intervals (categories) is that some intervals may contain very little data while others are well represented in the analyzed data set [40]. To compensate for this imbalance and create a more homogeneous classification, exposure levels with sparse presence of data should be represented by a wide interval of density estimation and vice versa. Ignoring this fact may mask possible contrasts between different exposure groups. In occupational literature using conventional EVA, exposure level categories have not been constructed with this purpose in mind [12,13,29,41], probably due to a concern for exploring exposure at suspected more hazardous levels rather than to optimize statistical performance [17]. However, a data-driven optimal exposure classification may reveal subtle changes in an exposure pattern, for instance due to an intervention, that will be left undetected by conventional methods [42]. The detection of such subtle changes may be relevant in studies of WMSD [8]. In particular for occupational work at low intensities and/or with repeated operations, minute changes in the exposure time pattern may be relevant. This wish to detect small changes in exposure, which would call for optimized data analysis procedures, is at conflict with the wish to keep the EVA lay-out constant across and within subjects so that inter- and intra-subject comparisons are possible. If data analysis is guided by the latter aim, EVA optimality cannot be achieved for all subjects and *exposure traces*. Securing commonality between subjects, e.g., in intervention studies, while still optimizing the classification performance of an analysis of exposure variation is a challenge for further research. A similar point can be raised if the results of C-EVA are compared between different studies because the optimal cluster centers may not match between the studies. However, if a major goal is to compare different groups or conditions, the gap analysis can be done for one of the conditions first, and the resulting cluster centers kept identical across the rest of the conditions.

Extending the number of representative exposure traces may improve the optimality of the method because it will improve the identification of the underlying exposure structure, but it may also result in lower generalizability of C-EVA as it may lead to over-fitting of the clusters to this particular structure.

In cases where exposure data are obtained from different sources in parallel, for instance in our simulations of one *exposure trace* comprising two *exposure realizations*, C-EVA provides one single outcome describing overall exposure variation whereas the conventional EVA would provide one result per source of data. This is an attractive property if data from different sources are correlated because a biased statistical analysis may be avoided by using a multivariate approach [43].

PCA was performed to identify a few components describing most of the variability in the data set. Thus, the classification was performed in a space, which had a lower dimension than the original one. An alternative approach could have been to implement a hierarchical regression model to assess exposure level, frequency and duration simultaneously, as suggested in a study by Jansen et al. [20].

In addition, the ratio between the number of observations and the number of variables is most likely larger in C-EVA than in a multivariate EVA, i.e., number of exposure traces in the training set divided by dimensionality of C-EVA and multivariate EVA marginal distributions ,i.e., (*n*_*c*_ *+ m*_*c*_*)* and 2(*n + m*), respectively. Thus, the PCA analysis of the output of C-EVA leads to more reliable outputs [44].

## Conclusion

The present study compared the abilities of a newly developed method, C-EVA, and the conventional EVA to discriminate patterns of exposure variation in simulated cyclic movements. The C-EVA has specific properties such as (i) a data-driven optimality of data reduction and (ii) the capability of handling multiple exposure time lines in one comprehensive analysis. The C-EVA slightly outperformed the conventional EVA in discriminating simulated *exposure traces* known to differ in variation, but both methods failed to detect differences in temporal similarity. The developed approach is promising in the sense that it furnishes a framework for assessing exposure variation, which is considered to be relevant in relation to development of work-related musculoskeletal disorders. Further research is, however, needed to develop methods that can sufficiently capture the similarity aspect of exposure variation and disentangle the trade-off between maximizing the information retained in the exposure data resulting from using an EVA-inspired approach and classifying exposure in a standardized set-up of categories assumed to be indicative of risks for musculoskeletal disorders.

## Abbreviations

ANOVA: Analysis of variance; C-EVA: Cluster-based exposure variation analysis; EVA: Exposure variation analysis; PCA: Principal component analysis; WMSD: Work-related musculoskeletal disorder.

## Competing interests

The authors declare that they have no competing interests.

## Authors’ contributions

All authors made substantial contributions to the conceptualization and writing of this manuscript. AS has implemented the method and is in charge of the integrity of the results. All authors read and approved the final manuscript.

## Authors’ information

AS has a background in biomedical engineering and science and now he is working as an assistant professor at the department of health science and technology in Aalborg University in Denmark. PM is the head of research interest group “Physical Activity and Human Performance” and director of the laboratory for Ergonomics and Work-related Disorders at the same institution. SEM is the research director of the Centre for Musculoskeletal research at the University of Gävle, Sweden.

## Pre-publication history

The pre-publication history for this paper can be accessed here:

http://www.biomedcentral.com/1471-2288/13/54/prepub
